# Identifying the ‘real cause of death’: the complexities of maternal death reviews in Tanzania

**DOI:** 10.1093/heapol/czag053

**Published:** 2026-04-20

**Authors:** Kerstin Almdal, Ali Said, Karen Marie Moland, Andrea Melberg

**Affiliations:** Centre for International Health, University of Bergen, Årstadveien 21, Bergen 5009, Norway; Department of Obstetrics and Gynecology, Muhimbili University of Health and Allied Sciences, 9 United Nations Road, Upanga West, P.O. Box 65001, Dar es Salaam, Tanzania; Centre for International Health, University of Bergen, Årstadveien 21, Bergen 5009, Norway; Centre for International Health, University of Bergen, Årstadveien 21, Bergen 5009, Norway

**Keywords:** MPDSR, maternal death, mortality review, cause of death, contributing factors, health systems

## Abstract

Identifying the causes of maternal deaths and contributing factors is essential for improving care. In 2015, Tanzania began implementing the maternal and perinatal death surveillance and response (MPDSR) system, including facility-based maternal death reviews. While most MPDSR studies highlight implementation and technical barriers, less is known about how systemic and institutional dynamics influence these reviews. This study examined stakeholders’ experiences and perceptions of MPDSR in Tanzania, focusing on how clinical causes of death and contributing factors were identified. The study is based on 5 months of ethnographic fieldwork conducted in a Tanzanian region in 2023–2024. It included 33 days of participatory observation of obstetric care, attending nine facility-based maternal death review meetings and conducting 20 in-depth interviews with health workers and administrative staff. Viewing MPDSR as a travelling model and drawing upon the concept of situated knowledge, we examined how institutional and professional factors influenced these reviews. Reviews were routinized and integrated into the regional health system, offering opportunities for teaching and defining standards of practice. However, participants disagreed on whether the reviews promoted quality improvement or focused on individual fault-finding, on how responsibility should be attributed, and whether reviews could accurately establish the causes of deaths. The facility-based death reviews were influenced by institutional and epistemic hierarchies, with responsibility often placed on individuals at the lowest health system level. While MPDSR aims to promote blame-free learning and quality improvement, the process narrowed attention to individual error, obscured systemic constraints, and hindered understanding of the ‘real cause’ of maternal deaths. To capture contextual complexity without adding reporting burden, we recommend expanding the free-text narrative fields in the official MPDSR maternal death report forms and increasing frontline representation in district- and regional reviews to strengthen links between facility and higher-level reviews.

Key messages

- Although MPDSR is formally integrated into the Tanzanian health system and the practice is routinized, many participants involved in the facility-based maternal death reviews felt that the focus was on individual fault-finding instead of being an opportunity for learning and quality improvement.
- The identification of causes of death and contributing factors occurring during facility-based maternal death reviews is influenced by institutional power and hierarchical positioning, with a tendency to attribute responsibility downward to lower-level facilities.
- There is a disconnect between the intention of MPDSR to promote accountability in a blame-free environment and the lived experience of frontline health workers, who reported a tendency to blame individuals.
- The official MPDSR death report forms require causes of death and contributing factors to be categorized into predefined boxes, which risks oversimplifying the multifaceted nature of events leading to maternal deaths.

## Introduction

Despite an estimated 40% decline in global maternal mortality since 2000, 700 women still die each day from pregnancy- and childbirth-related causes (World Health Organization 2025). Of these deaths, 70% occur in sub-Saharan Africa (SSA), reflecting the unequal burden of maternal mortality worldwide (World Health Organization 2025).

Maternal deaths result from both clinical causes and contributing factors. Clinical causes, medical conditions, or complications that initiate events leading to death or exacerbate an existing clinical state are commonly classified using the International Classification of Diseases for Mortality and Morbidity (ICD-MM) (World Health Organization 2012). In SSA, the most common clinical causes of death are obstetric haemorrhage, indirect obstetric causes, such as worsening of non-communicable diseases during pregnancy, hypertensive disorders, and abortion complications (Cresswell et al. 2025). However, clinical causes alone do not explain why maternal deaths occur. Contributing factors, including delays, contextual and patient-related issues, and system or referral weaknesses, can transform manageable conditions into fatal outcomes (Merali et al. 2014, World Health Organization 2025).

These contributing factors are conceptualized through various frameworks. The most widely used is the three delays model, which focuses on delays in seeking care, reaching the right facility, and receiving appropriate and timely care (Thaddeus and Maine 1994). Other frameworks include the availability, accessibility, acceptability, and quality (AAAQ) framework, which emphasizes structural barriers to care; the broader socio-ecological model, which adds organizational, community, and societal perspectives; and the clinically focused emergency obstetric care signal functions (Thaddeus and Maine 1994, World Health Organization 2015, Hadush et al. 2020, Muriithi et al. 2022). This diversity reflects the complexity of contributing factors and the need for different lenses to fully capture the actors, health system levels, and social and political dynamics involved (Bukenya and Golooba-Mutebi 2020, Muriithi et al. 2022).

Improving the identification of clinical causes and contributing factors leading to maternal deaths was one of the goals when WHO and partners launched the first technical guidance for the Maternal Death Surveillance and Response (MDSR) system in 2013 (World Health Organization 2013). This system functions as a continuous cycle of identifying, reviewing, planning, and, most crucially, responding to all maternal deaths (World Health Organization 2004, 2021). In 2016, perinatal deaths were included in what is now known as the MPDSR system (World Health Organization 2016, Kinney et al. 2021). Unlike earlier programmes, MPDSR should be integrated at all levels of the health system, with active surveillance, a mandatory response to every maternal death, and a commitment to promoting accountability in a blame-free environment (World Health Organization 2021, Whiting-Collins et al. 2024).

MPDSR unfolds within complex political, social, and institutional contexts as it operates across national, subnational, facility, and community levels. MPDSR includes various activities across levels of the health system. One key component of the system is the facility-based maternal death reviews, where the causes of maternal deaths are established. However, important barriers to these facility reviews have been reported in the context of MPDSR in countries such as Ethiopia, South Africa, Ghana, and Benin. These include weak governance, poor data quality, limited training, and insufficient resources (Kinney et al. 2020, Boyi Hounsou et al. 2022, Compaore et al. 2022, Willcox et al. 2024). In addition, several studies from SSA have documented a widespread fear of blame among health workers. This fear has been linked to the withholding or altering of information and the attribution of death to vague or external factors (Melberg et al. 2019, Said et al. 2020, Kidanemariam et al. 2024, Molenaar et al. 2025).

Tanzania was among the first countries to implement MPDSR as part of a political commitment to improve maternal health (Ministry of Health and Social Welfare 2015). With a population of ∼68 million, it is one of the fastest-growing nations globally and has a fertility rate of 4.6 children per woman (United Nations Department of Economic and Social Affairs 2024). According to UN inter-agency estimates, the maternal mortality ratio in 2023 was 276 maternal deaths per 100 000 livebirths (World Health Organization 2025).

In recent decades, Tanzania has introduced a broad range of maternal health policies and programmes (Ministry of Health Community Development Gender Elderly and Children (MOHCDGEC) 2021). Since 2015, MPDSR has been implemented countrywide, replacing earlier fragmented review programmes (Said et al. 2021b). Despite political commitment to MPDSR, studies from Tanzania report barriers similar to those in other countries, including poor data quality and uncertainty about roles and responsibilities. Limited training and time for reviews, inadequate tracking of response plans, and fear of blame further hinder implementation (Armstrong et al. 2014, Kashililika and Moshi 2021, Said et al. 2021a, Rumbeli et al. 2023).

The challenges reported regarding MPDSR reflect the problem of implementing universally designed programmes in resource-constrained settings (Melberg et al. 2019, Boyi Hounsou et al. 2022). As De Sardan (2018) argued, such programmes can be understood as travelling models, that is, internationally developed programmes standardized for implementation in any context. However, they often fail to sufficiently account for the complexities of the local context (De Sardan et al. 2017). According to De Sardan, context can be divided into structural context, which includes national policies, health system structures, and legal frameworks, and pragmatic context, which refers to local realities and everyday interactions (De Sardan 2018). While the structural context sets the conditions for implementation, the pragmatic context influences the practical function. Thus, the analysis of facility-based maternal death reviews requires an examination of both contexts and how actors navigate them.

The facility reviews mobilize a broad range of actors, each with distinct training, experience, and organizational identity, which influence their knowledge and perspectives. According to Haraway (1988), knowledge is neither universal nor objective. Thus, actors positioned differently in the health system produce situated knowledge (Haraway 1988), which influences how they experience and perceive the facility review process. In addition, power asymmetries influence how knowledge is produced and the type of knowledge perceived as legitimate or as holding epistemic authority (Michaels 2021).

While most MPDSR research has focused on implementation and technical barriers, fewer studies have examined how systemic, institutional, and contextual dynamics influence the facility review process itself (Willcox et al. 2020, Kinney et al. 2021). Given Tanzania’s decade-long experience with facility-based maternal death reviews, official political commitment to reducing maternal mortality and documented challenges surrounding the review process, it presents an important setting to explore these dynamics. This study aims to explore how different stakeholders experience and perceive the facility-based maternal death review process, with a particular focus on how causes of death and contributing factors are identified and attributed.

## Materials and methods

### Study setting

This study was conducted in a Tanzanian region with a population of ∼3 million people distributed across multiple districts. To secure participant confidentiality, the region has been deidentified. Approximately 60% of the population lives in rural districts, while 40% lives in and around the urban district in which the region’s capital city is located (The United Republic of Tanzania 2022). The region’s healthcare system follows the national referral pyramid structure, with dispensaries at the lowest level, followed by health centres, district hospitals, regional referral hospitals, and national hospitals (MOHCDGEC 2016). In Tanzania, low-risk pregnancies are managed at dispensaries or health centres, while high-risk pregnancies are referred to higher-level facilities (Ministry of Health Community Development Gender Elderly and Children (MOHCDGEC) 2021).

#### Facility-based maternal death reviews in Tanzania

Tanzania has adapted the World Health Organization’s MPDSR guidelines, which outline each step of the MPDSR cycle [Ministry of Health Community Development Gender Elderly and Children (MOHCDGEC) 2019]. According to these guidelines, a review meeting must be held within 7 days of a maternal death at the facility where the death occurred. These meetings are led by the facility’s MPDSR committee, which is composed of clinical staff, facility and district administrators, and support services personnel, depending on the facility level [Ministry of Health Community Development Gender Elderly and Children (MOHCDGEC) 2019]. According to the guidelines, a patient summary is presented, and the meeting participants assess the clinical causes of death, the contributing factors, and whether the death was avoidable [Ministry of Health Community Development Gender Elderly and Children (MOHCDGEC) 2019]. Findings should be recorded in the official Maternal Death Reporting Form, as well as in a document that sets out proposed, actionable recommendations, specifies responsible parties, and establishes timelines [Ministry of Health Community Development Gender Elderly and Children (MOHCDGEC) 2019]. These two documents are then submitted to the district and regional medical offices, thereby initiating the response phase of the MPDSR cycle.

### Fieldwork

This explorative qualitative study was conducted during two 3-month fieldwork periods in 2023 and 2024. Fieldwork included participatory observation in a tertiary obstetric department, attendance at the department’s MPDSR meetings, visits to various health facilities and 20 individual interviews.

As the first author and a Scandinavian medical doctor trained in obstetrics, I conducted 33 days of participatory observation at an urban tertiary teaching hospital during the spring months of 2023 and 2024. I followed the daily routines in the maternity ward and attended nine facility MPDSR meetings to understand clinical practice and the management of maternal complications and deaths. While not involved in clinical work, I joined clinical discussions and assisted with minor procedures. As English was the facility’s working language, I could follow ward rounds and meeting discussions. While I could speak basic Swahili, more complex discussions were conducted in English. To gain a broader view of maternal care across facility levels, shorter facility visits were conducted at a health centre, a district hospital, a regional hospital, and an outpatient clinic. Observational notes were recorded daily in notebooks and used for analysis and interpretation.

In addition, 20 individual semi-structured interviews were conducted, comprising six participants in administrative or leadership roles and 14 health workers. The administrative group included three administrative workers from regional and district offices and three health facility managers. The health worker group consisted of three health workers with formal MPDSR roles, two senior specialists, three residents from tertiary facilities, three doctors from district hospitals and health centres, and three nurses from a tertiary facility, a district hospital, and a health centre. This diverse group was recruited to gain a broad range of perspectives on the facility review process. An interview guide was developed, piloted, and revised throughout the study to explore participants’ experiences with maternal care and deaths, the facility review process, and perceptions of accountability and blame. Interviews were conducted in English and allowed for the exploration of topics as they emerged. In a few cases, a Swahili-speaking research assistant was present to provide translation. All interviews took place behind closed doors at the participants’ workplaces and lasted between 45 and 100 minutes. All but one was recorded and transcribed verbatim.

### Data analysis

Analysis of the observational notes and transcribed interviews was guided by Braun and Clarke’s reflexive thematic analysis approach, which acknowledges the researcher’s interpretive role and the contextual nature of the findings (Braun et al. 2014, Clarke and Braun 2017). Initial codes were developed using NVivo software and grouped into categories. Themes were then developed and refined through an iterative process, moving between the research aim and the data. From the onset of our analysis, we viewed MPDSR as a travelling model, distinguishing the structural context from the pragmatic reality experienced by participants. In framing and refining the themes, we were informed by Haraway’s concept of situated knowledge, especially when examining how positionality and epistemic authority influenced participants’ perspectives.

### Ethics

Ethical clearance was granted by the authors’ institute and the National Institute of Medical Research, and relevant regional, district, and health facility authorities. Research permits were issued by the Tanzania Commission for Science and Technology, and relevant regional, district, and health facility authorities.

Written consent was obtained from all interviewees. During the observation, verbal consent was obtained after explaining the study’s objectives and the participants’ right to opt out. While I did not continuously remind the participants of my role as a researcher to maintain the authenticity of the setting, I aimed to uphold ethical transparency by providing information whenever they enquired (Roulet et al. 2017). As a Scandinavian researcher, I remained aware that my outsider status may have influenced what participants shared. To address this, I engaged in ongoing reflexive discussions with co-authors and local stakeholders and strove to triangulate findings across interviews, observations, and informal conversations. When writing, potential identifying characteristics were carefully masked to strengthen confidentiality.

## Results

During the fieldwork, I observed several facility review meetings at the teaching hospital. Meeting participants included senior and junior obstetric doctors, senior midwives, MPDSR officials, and representatives from hospital administration and the regional office. Depending on the case, staff from other departments or health facilities also attended. Each facility meeting I observed followed the same structure. A junior doctor presented a patient summary using presentation slides, through which the woman’s care trajectory and the events leading to her death were described. In several referral cases I observed, staff members from the referral facilities presented their own summaries. The subsequent discussion was led by the MPDSR chair and focused on identifying the clinical cause of death, the contributing factors, and whether the death could have been avoided. Although the chair encouraged participation from all present, most attendees remained silent observers, and the discussion was mainly held among senior doctors, with occasional input from senior nurses or obstetric residents. When details were unclear, such as the timing of, or who performed, specific interventions, the presenting doctor or staff members involved in the patient’s care were asked to provide information, and they were sometimes criticized if they could not provide an answer. Each facility meeting concluded with the completion of the MPDSR death report form and the development of an action plan. A junior doctor would always read the form aloud, listing examples of ICD-MM codes for the clinical cause of death and possible subcategories under the ‘three delays model’ for the contributing factors. The action plan was documented by the MPDSR secretary as part of the meeting minutes. Projected onto a screen for all participants to see, each contributing factor (here noted as gaps) was listed along with recommended actions, the responsible parties, and an expected timeline.

Through analysis of the data material, it became apparent how the facility review process was integrated into the health system and how it served multiple purposes beyond reviewing maternal deaths. At the same time, the facility-based review process served as a site where different health system perspectives collided. The table below depicts the themes and sub-themes which will be further developed in the following (Table 1).

**Table 1:** Overview of analysis themes and subthemes.

Themes	Subthemes
Facility-based maternal death reviews as an integrated practice serving multiple functions	An integral part of hospital life
	Connecting the health system
	Facility review meetings as a learning arena
	Reinforcing the standard of care
Facility reviews as a site where perspectives collide	Quality improvement or fault finding?
	Tense relationship between higher- and lower-level facilities
	Conflicting views on the cause of death and contributing factors

### Facility-based maternal death reviews as an integrated practice serving multiple functions

#### An integral part of hospital life

Across all health facilities, MPDSR activities, and more specifically, facility-based maternal death reviews, were described as integral parts of hospital life. Participants at all levels demonstrated strong knowledge of the MPDSR programme and would at times correct me on timelines or regulations during interviews. All participants had firsthand experience with facility-based maternal death reviews. Some oversaw them administratively, while others participated as specialist advisors, general attendees, or as individuals who had been reviewed after treating women who later died. Regardless of their role, every participant could describe the different steps of the review process and considered the reviews a necessary response to each maternal death:

The focal person gathers information from the staff and relatives. From there, we arrange the meeting … for the MPDSR team and the people who were on shift when the death occurred. We engage in discussions, identify gaps, put in place an action plan, and work to prevent other deaths. (Nurse, lower-level facility)

While the structure of the facility review process was consistent across facilities, the frequency of reviews and the demands on staff time varied. In regional or teaching hospitals, facility reviews were held every week or every other week on a fixed day and time. Facility reviews in district hospitals and health centres were held less frequently because of fewer maternal deaths and were described by participants as special events. Administrative workers from district or regional health offices described how MPDSR took up a significant part of their work and included tasks such as overseeing facility review meetings, participating in district or regional MPDSR meetings, and tracking response plans. As one regional administrator described: ‘We visit the facilities to make sure that what they wrote in the report is really implemented on the site. We follow up with a regional report on the implementation status of the last resolutions’.

#### Connecting the health system

Rather than being isolated events, participants described the facility reviews as system-wide processes that extended beyond the facility where the death occurred, including all facilities where care had been provided. As described by a senior doctor, the information would flow upstream to district and regional offices: ‘Information [on the death] will be linked to the district, the region … all the way up to the ministry level’. At the end of all facility review meetings, I observed that the MPDSR secretary would take the MPDSR death report form and forward it to the administrative offices along with the action plan. According to the administrative staff, the information from the report forms and action plans served as the basis for discussions at district and regional MPDSR meetings and guided planning for the MPDSR response component. As reflected in the following quote, most participants—regardless of position—viewed this flow of information as necessary:

We discuss and then come up with resolutions, … not just as an isolated event, because we need to work in an intertwined mode. … We need to communicate and collaborate with the next district … with the region. We address all these matters together to achieve results. No one can just stand alone. (Administrator, regional office)

Many women who died at higher-level facilities were referred from those at a lower level. When reviewing these deaths, staff from all the facilities involved was invited to attend meetings held at the facility where the death occurred. Participants noted that this had previously required long travel and leaving care duties to overburdened colleagues. However, recent changes have made online attendance possible despite challenges such as unstable internet connections or suboptimal equipment. As noted by a facility administrator: ‘I think Zoom meetings have helped because people from outside the facility with different experiences and perspectives get to see the case and judge what happened’. In most facility review meetings I attended, staff from the referring facilities participated online by Zoom. In a few cases, they joined in person, ranging from a single junior doctor in one meeting to a larger group of eight participants in another, including administrative staff and health workers.

#### Facility review meetings as a learning arena

Administrative staff, department heads, and some health workers described the facility review meetings as valuable learning opportunities. They highlighted how discussing maternal deaths allowed health workers to learn about different medical conditions and ways of managing complications. Health workers from higher-level facilities noted that a key advantage was the presence of specialists at the MPDSR meetings, who contributed valuable knowledge and experience. As one junior doctor from a higher-level facility noted:

Everything’s here [regional hospital]: the investigation, every department, the pharmacists, lots of medications. … Meetings here are very lively compared to the [lower-level facilities]. … It inspires you to learn. And it’s like you are learning every day. Every death might have a common cause, but you find new things when you discuss them. (Junior doctor, regional hospital)

During facility review meetings at the teaching hospital, I saw senior specialists questioning junior health workers about their knowledge of diagnoses, treatments, outcomes, and what could have been done differently. If knowledge gaps were identified, specialists used the meetings to provide small lectures on specific aspects of care. Participants from lower-level facilities described how specialist obstetricians attended facility review meetings to provide technical support. As these facilities were usually staffed only with generalist doctors, the presence of obstetricians helped them better understand the chain of events leading to maternal deaths.

#### Reinforcing the standard of care

The participants described how reviewing each maternal death functioned as a mechanism for reinforcing the standard of care. During the observed meetings, discussions often focused on instances in which treatment or care had not adhered to established guidelines or standard operating procedures (SOPs). Health workers, however, admitted that different guidelines and SOPs existed, and some expressed uncertainty about which ones to follow. During visits to various health facilities, different SOPs were displayed on the walls of maternity units, originating from the Ministry of Health (MoH) or from research or quality improvement projects. Doctors from lower-level facilities also described how they received updated MoH guidelines from clinical WhatsApp groups. Amid this diversity of guidance tools, the facility review meetings served as an arena where ‘the proper standard of care’ could be clarified and reinforced. As explained by one administrative worker:

We are supposed to discuss, to have an MPDSR meeting, … what have you done? What is the best practice? (Administrative worker, district office)

### Facility reviews as a site where perspectives collide

#### Quality improvement or fault finding?

While participants agreed that facility reviews should occur in a blame-free environment that focused on quality improvement, their experiences varied. Administrative workers and MPDSR chairs emphasized that facility reviews were conducted for ‘the sake of improving care’ and described efforts to avoid placing unjust blame. They highlighted the guideline motto, ‘No name, no blame’, which MPDSR chairs stated at the beginning of each meeting to ensure ‘good and open communication’. I frequently observed the chair reminding participants that the meetings were not ‘courtrooms’ but learning spaces. Some administrative workers stressed that even if individual mistakes were identified, blame should not be the focus of the facility reviews:

In MPDSR, we have a saying: ‘No blame, no shame.’ It is the slogan. We are not supposed to sue someone—that ‘you are responsible for that death’. Even though in the discussion, when you are going through the case, … you will find where it went wrong and who was wrong. But we should not blame. We are just providing an education. (Administrative worker, district office)

Generally, participants avoided using terms such as ‘error’ or ‘mistake’, even though most maternal deaths were considered avoidable. It was notable how most participants preferred neutral terms, such as ‘gaps’, ‘issues’ or ‘weaknesses’. They often used impersonal phrasing such as ‘the major gaps were [related to] lack of knowledge’ or ‘the issue is a lack of skills’, rather than blaming individuals.

Several participants articulated a strong commitment to the ‘no name, no blame’ principle, yet later described instances in which they had assigned individual blame. They described how this was warranted when someone acted below the expected standard of care. As a participant working in a district administration office noted, ‘We’re always looking for something that was not done so that we can take action against that person’.

This duality was also recognized by frontline health workers, who were familiar with the no-blame principle but perceived it as a largely symbolic statement that did not translate into practical implementation. As a senior obstetrician noted:

Death is a complex thing, so we should examine each component of the system, but most of the time, we end up blaming the health provider. (Specialist doctor, higher-level facility)

#### Tense relationship between higher- and lower-level facilities

During the facility reviews, disagreements emerged between staff from higher- and lower-level facilities regarding the assessment of clinical causes of death, contributing factors, and where responsibility should be assigned. Some participants argued that the contributing factors should be attributed to the lower-level facilities where they emerged, while others argued that the responsibility lies with higher-level facilities where the death ultimately occurred.

Generally, participants agreed that higher-level facilities had more resources and management options. However, staff at lower-level facilities reported being criticized during facility reviews for inadequate care and delayed referrals, and being expected to handle surgeries, blood transfusions, and complicated births despite limited capacity. As one district doctor explained:

When patients visit higher-level facilities, most health workers from the high centre do not recognize the effort put in by the lower centre. They always find gaps and weaknesses. Our environment [secondary level] differs from their environment [tertiary level]. … So always, when you refer the patient to the high centre, … they are so harsh. (Doctor, lower-level facility)

The timing of referrals was another source of disagreement between facilities at different levels. According to participants’ accounts and my observations, many women arrived at higher-level facilities in a critical condition, often too late for effective treatment. When these deaths were reviewed, participants often disagreed about whether the referral had been appropriately timed and where responsibility for the death should lie. Lower-level health workers reported being criticized for referral timing and being held responsible for subsequent deaths, regardless of circumstances, with some cases being sent to district disciplinary committees.

Higher-level staff argued that many women were either referred without a proper clinical indication, occupying space needed for critically ill patients, or referred much too late—often in near organ failure—when survival was unlikely. They emphasized that in these ‘late referral’ cases, the clinical cause of death and contributing factors had already developed at the sending facilities, making tertiary management highly challenging. As one senior obstetrician from a tertiary facility noted, ‘If we only focus on our own facility, … the vegetative patients keep on coming’; therefore, ‘we should be able to look downwards [to lower-level facilities]’.

#### Conflicting views on the cause of death and contributing factors

There were conflicting views on whether facility reviews could properly reconstruct the events leading to maternal deaths and identify accurate causes of death and contributing factors. Those with formal MPDSR roles argued that it was possible to conduct thorough facility reviews. In contrast, frontline health workers were more inclined to emphasize the complexity of multifaceted care trajectories and questioned whether it was possible to identify the ‘real cause of death’.

All participants agreed that proper reviews require comprehensive information, yet many pointed out that this information was often lacking. One doctor described most facility reviews as having ‘poor documentation’, lacking vital information about patients’ conditions, treatment provided, timing of interventions, and reasons for delays. Several participants highlighted short referral letters as a major barrier. When documentation was missing, health workers were asked to recall the care they had provided. However, as one lower-level doctor explained, it was often difficult for one person to remember the care provided by several colleagues. I observed these challenges at several facility review meetings, where insufficient documentation made it difficult to assess what had happened.

Participants in administrative roles argued that the main challenge in accurately identifying the cause of death and contributing factors was not case complexity but health workers’ reluctance to describe in detail the events leading up to the death. An MPDSR chair described the need to ‘probe sufficiently’ during facility reviews. Echoing this, an MPDSR focal person stated that when meetings were conducted ‘properly’ and people ‘talked openly’, finding the ‘real cause of death’ was ‘not difficult’. Health workers with managerial responsibilities often bridged these opposing perspectives. They acknowledged the complexities of care trajectories and review limitations while maintaining that the correct causes and contributing factors were usually accurately identified and led to effective action plans.

Health workers highlighted that establishing a precise link between the clinical cause of death, contributing factors, and death was challenging. Many expressed that the contributing factors were often not sufficiently investigated, with facility reviews focusing mainly on the medical diagnosis, as reflected in the quote below:

It [the facility reviews] will not identify the full explanation for the deaths. You won’t be able to prevent the next one because you don’t know what caused it. Does every woman with eclampsia die? No. So why did this patient die? We cannot just conclude that the patient died because of eclampsia. Why did the other patient with eclampsia survive? (Junior doctor, higher-level facility)

Health workers frequently pointed out that the structural issues underlying maternal deaths were rarely examined in depth. Recurrent examples included lack of oxygen tanks for referrals, blood for transfusion, and qualified personnel. Doctors from lower-level facilities told stories of newly qualified doctors with little surgical training being needed to perform complicated procedures, such as caesareans or hysterectomies, because they were the only doctors available. Most emphasized that framing these issues as human error and sending staff for additional training would not address the underlying constraints in resources, surgical training or supervision.

## Discussion

This study of the facility-based maternal death reviews in a Tanzanian region has documented how these facility reviews were routinized and integrated into the regional health system, serving as opportunities for teaching and defining standards of practice. The facility reviews also became sites where participant positionality and power hierarchies came into play when perceptions about the causes of maternal deaths collided. In the following section, we discuss the contextual challenges of facility reviews, how these reviews mobilize inherently situated knowledge, and lastly, the implications of how causes of maternal deaths are established.

### Contextual challenges for facility-based maternal death reviews

MPDSR is an example of a travelling model—a standardized programme developed internationally to be implemented locally with minimal adaptation (De Sardan 2018, Melberg et al. 2019). Our findings showed that the facility reviews appeared well supported by what De Sardan coined as the ‘structural context’ through national MPDSR policies, integration into the health system, and institutional arrangements such as reporting structures and established committees. Guidelines were clearly defined, facility review meetings were held regularly, and findings were consistently documented for each facility review process. Nevertheless, the study participants experienced a disparity between the intended function and the practical realities of facility reviews. In De Sardan’s terms, this represented a disconnect between the structural and pragmatic context (De Sardan 2018). While MPDSR’s official aim is to gain ‘a meaningful understanding of the events’ and ‘not to find fault or discipline providers’ (World Health Organization 2021, p. 25), participants reported major barriers, including insufficient documentation, fragmented care across facilities, fear of blame, and avoidance of speaking up.

The perception of facility reviews as a fault-finding mechanism contrasts with MPDSR’s motto of ‘no blame, no name’ (Ministry of Health Community Development Gender Elderly and Children (MOHCDGEC) 2019). While higher-level staff claimed that these principles were upheld, frontline health workers experienced them as largely symbolic. This aligns with studies from Ethiopia, South Africa, Benin, Nigeria, and Indonesia, where fear discouraged disclosure of the care provided (Melberg et al. 2019, Kinney et al. 2022b, Said et al. 2021b, Boyi Hounsou et al. 2022, Cetin et al. 2022). In our study, the fear of disclosing details about the chain of events leading to death appeared to increase the difficulty of identifying the clinical causes of death and contributing factors.

In addition, widespread documentation challenges were revealed. Despite national efforts to improve the medical record system (Ministry of Health Community Development Gender Elderly and Children (MOHCDGEC) 2019), participants reported that the information required to conduct proper facility reviews was frequently missing. This aligns with findings across SSA, where insufficient data have been linked to an increased risk of misdiagnosing the cause of death (Mgawadere et al. 2016, Musarandega et al. 2021, Willcox et al. 2024). While facility reviews appeared to be formally well organized, the practical context posed considerable barriers to accurately identifying and attributing the cause of death and contributing factors. Acknowledging and addressing barriers emerging from the practical context is vital to bridge the gap between the intended function and the practical realities of the facility reviews. As illustrated in our findings, health workers with dual positions have the potential to take a leading role in such processes.

### Situated perspectives on the review process

Participants’ experiences and perceptions of the review process varied according to their clinical experience, hierarchical position, and MPDSR involvement. These differences reflect Haraway’s (1988) concept of situated knowledge, in which participants’ education, professional role, and lived experiences influence their perceptions and their authority to produce knowledge (Haraway 1988).

Three intersecting dimensions appeared to shape these diverging perspectives: embodied experience gained through direct patient care, hierarchical position, and formal MPDSR roles providing specific training and knowledge of the MPDSR programme. The most significant contrast emerged between health workers from lower-level facilities and district- or regional-level administrators. However, health workers at all levels who were involved in patient care and making treatment decisions emphasized both fear of blame and the complexities of identifying causes of death and contributing factors. In contrast, district and regional administrative workers who were not involved in clinical practice, but organized care delivery and allocated resources viewed MPDSR primarily as a quality improvement tool. They described being answerable to the MoH and appeared to focus on developing feasible responses to maternal deaths and demonstrating visible results to stakeholders higher up in the healthcare hierarchy. Similar dynamics were observed in a study in Burkina Faso regarding a mobile health programme. There, health workers were concerned with the practical challenges of the programme and its effect on patient care, while administrative workers were more focused on project coordination and outcomes, reflecting their different responsibilities and situated knowledge (Duclos et al. 2017).

Our study also included participants who bridged the contrasting perspectives described above. Their dual perspective seemed to be linked to them navigating between the personal experiences of providing direct patient care and the administrative demands of being answerable to higher-level administrative offices. Such dual positions have been linked to enhanced implementation of health system policy elsewhere (Hirvonen 2024). Health workers with both clinical practice and administrative training, as well as a thorough understanding of the organizational and political context within which they worked, were key factors in the implementation processes (Hirvonen 2024). Similarly, we argue that enhanced understanding of, and dialogue with, the processes at higher levels and the MPDSR system as a whole would make clinicians better equipped to implement the reviews as intended. As such, closing the gaps between the levels of MPDSR system implementation is the key to the overall functioning of the system.

The forms of knowledge mobilizing institutional authority have the greatest opportunity to influence the decisions made in a healthcare context (Haraway 1988, Hirvonen 2024). However, perspectives providing the most critical insight are often from lower levels in the hierarchy, as they are ‘least likely to allow denial of the critical and interpretive core of knowledge’ (Haraway 1988, p. 583). The results reflected this tension: clinically experienced participants had valuable knowledge but lacked access to decision-making spaces or the authority to voice their opinions. In contrast, higher-level administrators held institutional power but had less clinical expertise. This dynamic likely contributed to the way causes of death and contributing factors were identified and attributed, indicating the influence of contextual and institutional elements. Similar dynamics were reported in facility-based MPDSR reviews in Uganda, where hierarchical dynamics and fear of blame shifted attribution towards individual rather than structural causes (Namagembe et al. 2022). We argue that exploring mechanisms to strengthen the epistemic authority of lower-level health workers in facility reviews would be valuable to ensure more locally actionable response plans.

### Identification and attribution of gaps

One of the most contested aspects of the facility reviews was the identification and attribution of contributing factors. Tanzanian MPDSR guidelines label these as ‘associated factors’ and organize them with the ‘three delays model’, while other national MPDSR guidelines and the wider maternal health literature use terms such as ‘non-clinical events’, ‘enabling factors’, or ‘dysfunctions’ and categorize them using other frameworks (Merali et al. 2014, Hussein et al. 2016, Ministry of Health Kenya 2017, Muriithi et al. 2022, Boyi Hounsou et al. 2024). Some countries have even developed their own frameworks, such as Nigeria’s ‘barriers to care and remedial factors’ (Federal Ministry of Health 2015). This diversity likely reflects how maternal deaths arise from multifactorial care trajectories with events linked together across health system levels (Madzimbamuto et al. 2014, Muriithi et al. 2022).

As in Tanzania, the three delay model is one of the most widely used frameworks for analysing maternal deaths (Pacagnella et al. 2012). Its popularity may stem from its clinical orientation, simple structure, and focus on the patient, community, and facility level. However, it has also been criticized for being too simplistic and sequential, lacking the complexity required to capture contextual or preventive factors (Danna et al. 2020). Based on our study findings, we argue that a single framework may not be sufficient to encapsulate the complex and multilayered nature of maternal deaths. Some argue that more than one framework is needed (Pacagnella et al. 2012, Muriithi et al. 2022), but we are cautious about introducing yet more forms and reporting requirements to health workers in resource-constrained settings where reporting may draw attention from clinical care (Melberg et al. 2018).

MPDSR reports with their predefined subcategories are intended to streamline facility review outcomes and make the complex care trajectories easier to understand and compare at a higher level to guide response. Therefore, they depend on clear terminology and frameworks for standardization. While standardization can help, it also risks oversimplifying and decontextualizing maternal deaths. These challenges align with De Sardan’s concerns regarding travelling models, of which MPDSR can be considered one. The official death report form is designed as an operational tool, simplified to facilitate its implementation, but this simplification risks decontextualization and fails to capture complexity. A study from Benin, for instance, reported how MPDSR facility-based maternal death reviews frequently identified ‘superficial’ factors, focusing on immediate, observable events rather than addressing deeper systemic issues (Boyi Hounsou et al. 2024). However, evidence from South Africa showed that well-implemented perinatal MPDSR audits can translate findings into concrete service delivery improvements (Kinney et al. 2022a). Lessons drawn from analysing the practical norms in such contexts could inform implementation strategies elsewhere.

Participants tended to use non-confrontational terms such as ‘gaps’ and ‘issues’, avoiding ‘errors’ or ‘mistakes. The use of these terms may stem from participants attending different MPDSR training courses, but it may also reflect a fear of being blamed for a perceived mistake. However, this non-confrontational language did not ensure a neutral attribution of contributing factors. Despite MPDSR’s emphasis on system-level improvements and ‘no name, no blame’, responsibility tended to be attributed downwards by those holding institutional and epistemic power to individuals or facilities lower in the healthcare system. Similar patterns were observed in Indonesia, where junior reviewers avoided criticizing senior doctors, thereby misattributing contributing factors to lower levels. In the United States, internal committees tended to report patient-related factors, while external committees more often identified provider- and system-level issues (Geller et al. 2015, Cahyanti et al. 2021). In South Africa, maternal death reviews tended to lead to fault-finding rather than proactive quality improvement, unlike perinatal or other review platforms, likely reflecting the political sensitivity surrounding maternal deaths (Mukinda et al. 2021).

Regardless of the terminology used, a persistent point of disagreement was whether the factors contributing to maternal deaths primarily occurred at the individual level or were broader systemic issues. Administrative workers often cited a ‘lack of knowledge and skills’ as the most common gap, whereas frontline health workers emphasized systemic issues, such as resource shortages or understaffing. This illustrates how systemic constraints can be reframed as individual faults when voiced by those with epistemic authority (Haraway 1988).

When facility-level identification of contributing factors travels upstream and is categorized as individual misconduct rather than structural constraints, it risks skewing the information available for higher-level reviews and limits their ability to identify system-level challenges. While MPDSR is intended to promote learning and quality improvement in a blame-free environment, the facility review process can narrow the understanding of systemic challenges, reinforce blame, and ultimately hinder a comprehensive understanding of the ‘real cause’ of maternal deaths.

## Limitations

Some limitations should be noted. First, the study context was a combination of urban and semi-urban settings and did not include participants who worked in rural facilities. Rural settings differ from urban areas in the availability of specialists, of distances, and of patient complexity. Despite efforts, it was also not possible to attend district or regional review meetings. Including these perspectives may have revealed additional barriers, such as higher rates of staff shortages, supply chain issues, and longer referral distances, which could have altered the results. Moreover, it was beyond the scope of the study to detail MPDSR processes of the aggregated deaths at subnational and national levels, which also need to be considered if we are to fully assess the causes of maternal deaths. Despite these limitations, we argue that the study provides valuable insights into the facility review process by combining observations and interviews, thereby capturing a broad range of perspectives from participants across facility and administrative levels.

## Conclusion

Although facility-based maternal death reviews are formally integrated into the Tanzanian health system, our findings show how the practical context of implementation and power dynamics influence these reviews and challenge their intended function. Designed to be a blame-free, system-wide learning tool, facility reviews were often experienced as contested arenas influenced by institutional roles, clinical seniority, and hierarchical positions. These dynamics can mask systemic issues and reinforce blame downwards, particularly onto lower-level facilities and frontline health workers. To strengthen facility reviews as a mechanism for quality improvement and learning, practical and systematic constraints must be addressed. To better capture the contextual complexity without adding reporting burden, we recommend expanding the free-text narrative fields in the official MPDSR maternal death report forms and increasing frontline representation in district- and regional reviews to reinforce links between facility and higher-level MPDSR processes. Future research should trace how data on causes of death and contributing factors travel from facility reviews to subnational and national levels, and how complex real-life events are translated into maternal mortality indicators and response plans within MPDSR.

## Data Availability

Data cannot be shared for ethical/privacy reasons.

## Reflexivity statement

We acknowledge that our professional roles, prior research experience and institutional affiliations have likely shaped this study. As researchers from Norway and Tanzania with training in clinical medicine, anthropology and social science, our perspectives shaped the study’s design, data collection, analysis and interpretation, including prioritizing certain aspects above others. We actively sought to reflect on these issues throughout the research process. This included presenting preliminary findings for discussion within internal research groups and engaging in conversations with Tanzanian collaborators. Given the sensitive nature of maternal deaths, we paid careful attention to ethical considerations, power dynamics and respect for participants and patients.
